# Dispersion of antimicrobial resistant bacteria in pig farms and in the surrounding environment

**DOI:** 10.1186/s42523-024-00305-8

**Published:** 2024-03-30

**Authors:** Daniel Scicchitano, Daniela Leuzzi, Giulia Babbi, Giorgia Palladino, Silvia Turroni, Cédric Christian Laczny, Paul Wilmes, Federico Correa, Pimlapas Leekitcharoenphon, Castrense Savojardo, Diana Luise, Pierluigi Martelli, Paolo Trevisi, Frank Møller Aarestrup, Marco Candela, Simone Rampelli

**Affiliations:** 1 Fano Marine Center, Fano, Italy; 2 Department of Agricultural and Food Sciences, University of Bologna, Bologna, Italy; 3 University of Luxembourg, Esch-sur-Alzette, Luxembourg; 4 Tecnhical University of Denmark, Kongens Lyngby, Denmark; 5 Department of Pharmacy and Biotechnology, University of Bologna, Bologna, Italy

**Keywords:** Microbiome, Antibiotic resistance gene, Resistome, Food safety, Swine microbiome

## Abstract

**Background:**

Antimicrobial resistance has been identified as a major threat to global health. The pig food chain is considered an important source of antimicrobial resistance genes (ARGs). However, there is still a lack of knowledge on the dispersion of ARGs in pig production system, including the external environment.

**Results:**

In the present study, we longitudinally followed one swine farm located in Italy from the weaning phase to the slaughterhouse to comprehensively assess the diversity of ARGs, their diffusion, and the bacteria associated with them. We obtained shotgun metagenomic sequences from 294 samples, including pig feces, farm environment, soil around the farm, wastewater, and slaughterhouse environment. We identified a total of 530 species-level genome bins (SGBs), which allowed us to assess the dispersion of microorganisms and their associated ARGs in the farm system. We identified 309 SGBs being shared between the animals gut microbiome, the internal and external farm environments. Specifically, these SGBs were characterized by a diverse and complex resistome, with ARGs active against 18 different classes of antibiotic compounds, well matching antibiotic use in the pig food chain in Europe.

**Conclusions:**

Collectively, our results highlight the urgency to implement more effective countermeasures to limit the dispersion of ARGs in the pig food systems and the relevance of metagenomics-based approaches to monitor the spread of ARGs for the safety of the farm working environment and the surrounding ecosystems.

**Supplementary Information:**

The online version contains supplementary material available at 10.1186/s42523-024-00305-8.

## Background

The spread of antimicrobial resistance through the planet microbiomes is a global concern that poses a risk to the entire biome, including plants, animals and humans, particularly for what concern the possible risk of horizontal-transfer to pathogens or potential pathogens, as passenger in the microbiome ecosystem [55]. Such resistance is generated by genes called antibiotic resistance genes (ARGs), which are present in microbial genomes and ensure survival when exposed to antimicrobial molecules. The spread of ARGs across the planet microbiomes can occur through several mechanisms, such as: (i) direct transmission of antibiotic resistant bacteria (ARB) between different ecosystems, (ii) horizontal gene transfer of ARGs between different microbiome components, as promoted by mobile genetic elements [10, 26], and/or vertical transmission process [27]. It is clear that, the overuse of antibiotics in anthropic systems in past years has favored the selection of ARB and ARGs, as well as their subsequent spread across all planet biomes, with animal farming being a hotspot of evolution and resistance spread [29]. For this reason, the use of antibiotics in animal farming was prohibited for growth promotion purposes in Europe in 2006, and since then antibiotics can only be used for veterinary purposes such as metaphylaxis, prophylaxis, and medication to protect animal wellbeing [30, 32].

Concerns about the spread and dissemination of ARB and ARGs to environmental biomes are relevant in the pig system, where antibiotic administration has historically been widespread, especially in the early stages of pig’s life [4, 5]. Indeed, a large multi-country study of 9 European countries showed that most antibiotics, especially penicillins and polymixins, are routinely administered to weaners (69.5% of total TIDDDvet, defined as the time a pig is treated with antibiotics) and then followed by suckling piglets (22.5% of total TIDDDvet [12, 45]). Piglets exhibit a pre-existing resistance pattern from birth that reflects their environment, encompassing resistance to tetracyclines, β-lactams, and aminoglycosides [7]. Despite that, a decrease in the abundance of antimicrobial resistance carried by these animals with age has been observed, irrespective of the geographic area [17]. For these reasons, pigs can act as reservoirs of ARB and ARGs throughout their lives, posing a significant health risk to the surrounding environment and the associated biome [32, 40]. To the best of our knowledge, the environmental spread of antibiotic resistance from pig farms is mainly due to the use of pig manure as a soil fertilizer, and the discharge of farm wastewaters into the environment, to fertilize soils prior to crop production [16, 32, 46]. Some studies, such as those by Teng et al. [51] and Gao et al. [19], showed up to 5 years of soil contamination with ARB when fertilized with “contaminated” manure. Residues of antibiotics and ARB have also been found in slaughterhouses, where animals arrive at the end of the production cycle, just before being placed on the market [46]. For all these reasons, it is becoming increasingly urgent to monitor the spread of antimicrobial resistance in the pig food production cycle in order to prevent environmental contamination and for the selection of more effective antibiotic therapy to keep animal health in the food production system.

In this scenario, and in the context of the project “Controlling Microbiomes Circulations for Better Food Systems” (CIRCLES, https://circlesproject.eu/), funded by the European Union’s Horizon 2020, we evaluated the presence and distribution of microorganisms and ARGs in two pig farming systems in Italy, in a longitudinal setting during a commercial production process. To this end, a food system metacommunity-based approach was implemented. The reconstruction of metagenome-assembled genomes (MAGs), from the food system microbiomes (i.e. fecal, air, boots, soil, water, wastewater and slaughterhouse environment) allowed the best resolution for ARGs assessment in the pig food system, tracking the circulation of species-level genomes and their associated ARGs from the pig gut to the internal and external farm environments and to the slaughterhouse. Our results indicate the dispersal of microorganisms and their associated antibiotic resistance genes from the pig food system to the external environment and even to the final stage of meat production, suggesting the importance of metagenomics-based community assessment for a systematic evaluation of the risks associated with the spread of antimicrobial resistance in this food system.

## Results

### SGBs-level characterization of microbiomes in the pig food system

**Fig. 1:**
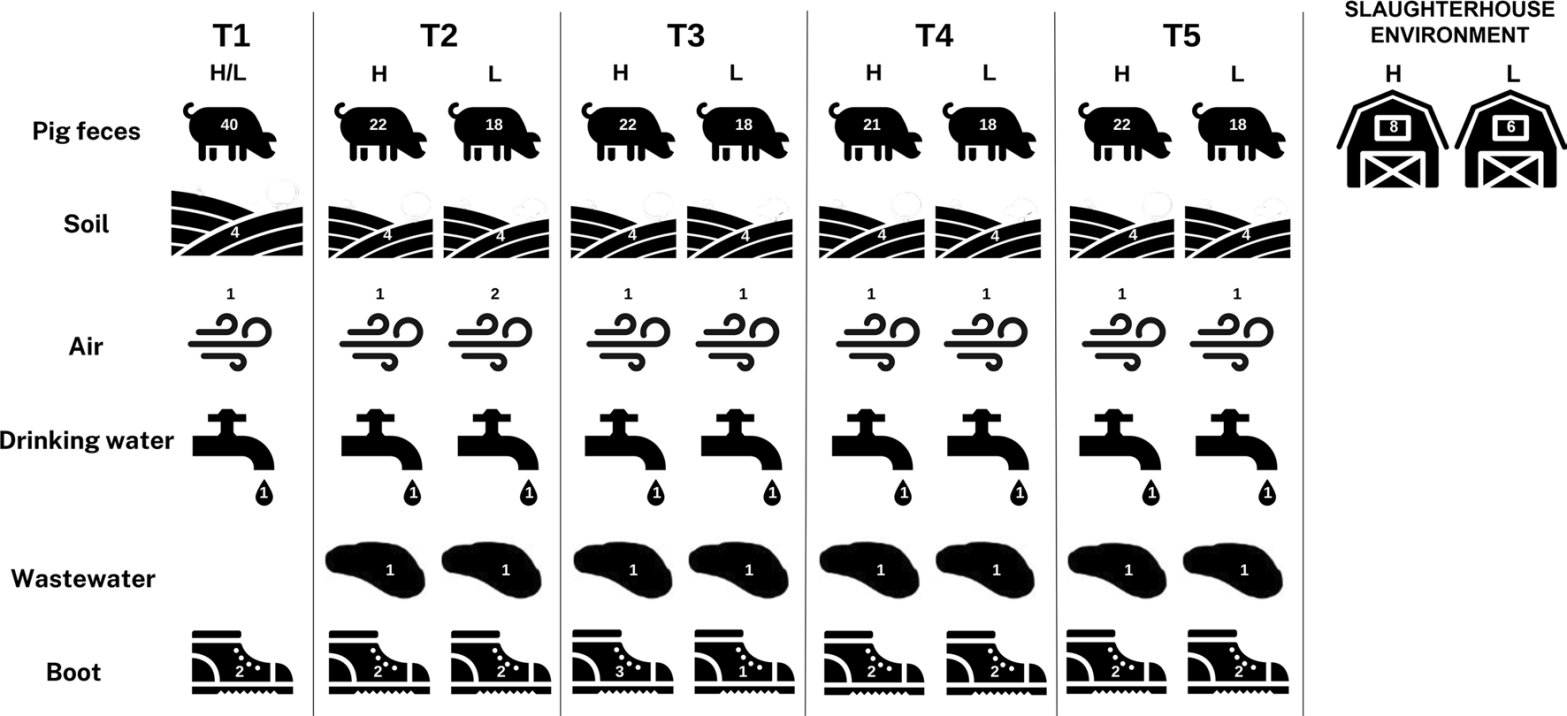
Schematic representation of the study design and the number of samples collected at each timepoint in each farm. The microbiome of pig and farm environmental samples (including pig gut, soil surrounding the farm, air inside the farm, animal drinking water, wastewater, workers boot soles and slaughterhouse environment) were collected at 5 different timepoints during the rearing cycle in two different Italian farms labeled as H & L. T1: farrowing unit, piglets from birth to 24 days; T2: start of the weaning phase; T3: end of the weaning phase; T4: start of the growing-finishing phase; T5: end of the growing-finishing phase. The numbers within the icons indicate the number of samples collected

A total of 294 samples were processed for DNA extraction and shotgun metagenomic sequencing. Specifically, two production chains originating from the same farrowing unit were sampled longitudinally at 5 timepoints, following the entire rearing cycle of two groups of pigs (see Additional file 4: Table S1). The two production chains consisted of a common farrowing unit, subsequently split into two weaning units and two final growing-finishing units. A total number of 199 fecal samples from pigs; 18 swabs from workers boots and 10 air samples from the farm, representing the internal farm ecosystem; and 9 water sample from the watering place, 8 wastewater sample from the lagoon and 36 soil samples from the surrounding area, representing the external farm environment. In addition, a total of 14 swab samples from various surfaces in the slaughterhouse were collected at the end of the rearing cycle and during the slaughter phase for each pig group. An overview of the study design and sampling during the pig production cycles is shown in Fig. 1 & Additional file 4: Table S2.

A total of 2.02 billion paired-end raw reads were generated, with an average of 6.9 million reads per sample. From the 294 metagenomes, we were able to reconstruct 2,704 high-quality MAGs, considering only those with more than 50% completeness and less than 5% contamination, with a total median per sample of 9 MAGs (median of MAGs based on sample type: 10 pig gut, 8 workers’ boot, 2 air, 8 water, 11 wastewater, 1 soil and 1 slaughterhouse ecosystems) and a binning efficiency of 40.47%. The MAGs were then dereplicated into 530 SGBs, considering 95% similarity as the minimum threshold for clustering MAGs together (see Methods section for further details and Additional file 4: Table S3). We then mapped these 530 SGBs against previously explored MAGs (> 7000) from the available pig gut microbiome [6, 20, 23] and the SGBs from > 150,000 human gut microbiome (GM) MAGs, including different individuals, spanning age, geography, and lifestyle [38]. In total, 367 SGBs (69%), clustered with at least one known reference genome (the full list of known SGBs is reported in Additional file 4: Table S4), while the remaining fraction of SGBs (163 SGBs, 31%), showed > 5% genetic distance to any SGBs of the databases and were considered as unknown genomes never detected before.

**Fig. 2:**
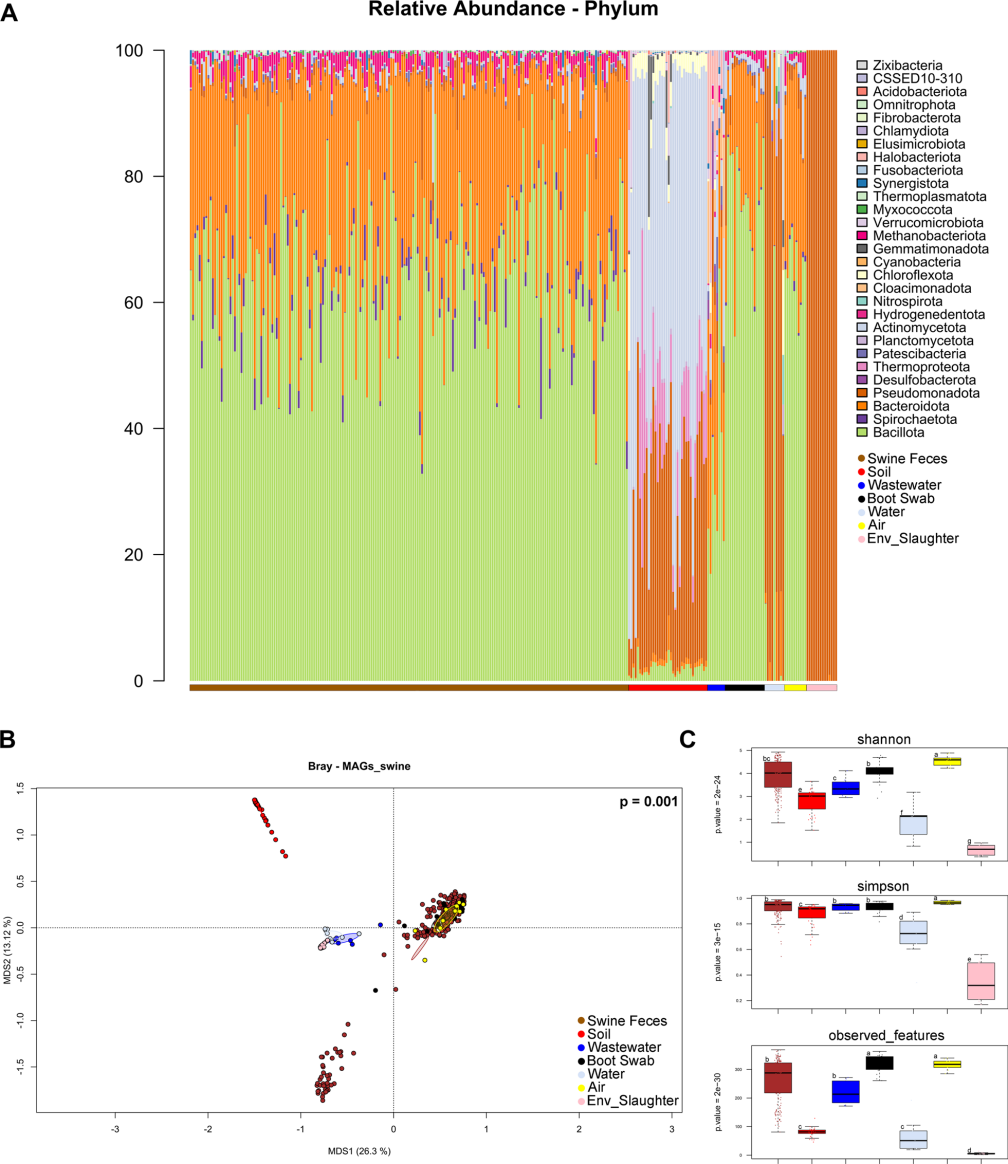
Composition of pig gut, farm environment, and slaughterhouse environment microbiomes. **A** Taxonomic classification of species-level genome bins (SGBs), represented as relative abundance at the phylum level across samples (pig gut microbiomes, soil surrounding the farm, air within the farm, animal drinking water, wastewater, workers boot soles and slaughterhouse environment). **B** Principal Coordinate Analysis based on the Bray Curtis distances between the SGB profiles of the different samples. The percentage of variance in the dataset explained by each axis is reported within the graph (permutation test with pseudo-F ratio, *p*-value = 0.001). **C** Alpha diversity boxplots based on Shannon index, Simpson index and observed features (number of SGBs). A statistically significant variation (Kruskal–Wallis test, *p*-value < 0.001) of alpha diversity among microbial ecosystems was found with all metrics. Sample groups marked with different letters are statistically significant different (Wilcoxon rank-sum test, *p* < 0.05). Sample groups are colored according to the color legend within the PCoA plot

After taxonomic profiling, 29 bacterial phyla were identified within the dataset of metagenomic samples (Fig. 2A), where we observed a similar profile in terms of most abundant phyla for pig gut microbiome (mean relative abundance, Bacillota 64%–Bacteroidota 29%), boots swabs (Bacillota 76%–Bacteroidota 18%), air (Bacillota 65%–Bacteroidota 26%) and wastewater (Bacillota 33%—Bacteroidota 27%), but with a different relative abundance. While, in the other ecosystems we observed a different microbiota profile, with soil samples dominated by Actinomycetota at 52% and Pseudomonadota at 28%, and water and slaughterhouse environmental samples dominated by Pseudomonadota at 71% and 99%, respectively. At the species level (SGBs), the pig gut microbiome was mostly dominated by *Lactobacillus amylovorus*, *Cryptobacteroides* sp000431015 and *Limosilactobacillus reuteri* (mean relative abundance, 14%, 4% and 3%, respectively). The samples from the internal farm environment (air, boot soles) showed almost the same most abundant species, such as *L. amylovorus* and *L. reuteri* (18% and 4%, respectively), with the addition of a known pig-derived species *Aerococcus urinaeequi* (4%) [33]. The microbiomes from the external environment (drinking water, wastewater and soil) were mainly dominated by f_*Nitrososphaeraceae;s_*TA-21 sp014523595, *Novosphingobium* sp015657645 and *Methanothrix soehngenii* (6%, 3% and 2%, respectively). On the other hand, the slaughterhouse environment was characterized by *Pseudomonas cremoris*, f_*Rhodocyclaceae*;s_SFHR01 sp004555545 and *Novosphingobium* sp015657645 (57%, 33% and 6%, respectively).

The SGBs microbiome structures in the two production chains, including those of the pig gut, the internal and external environment, and the slaughterhouse, were then compared by principal coordinates analysis (PCoA) using the Bray–Curtis distance (Fig. 2B). According to our results, the different ecosystems clustered separately, regardless of the farm (permutation test with pseudo-F ratio, *p*-value = 0.001) except for the air and boot sole samples, which showed an almost comparable microbial layout (*p* > 0.05). Focusing on alpha diversity, we observed significantly lower values in the microbiome of soil around the farms, water and slaughterhouse samples compared to the other ecosystems (Wilcoxon rank-sum test, *p* < 0.05) (Fig. 2C).

### SGBs potential dispersion across the farm system

**Fig. 3:**
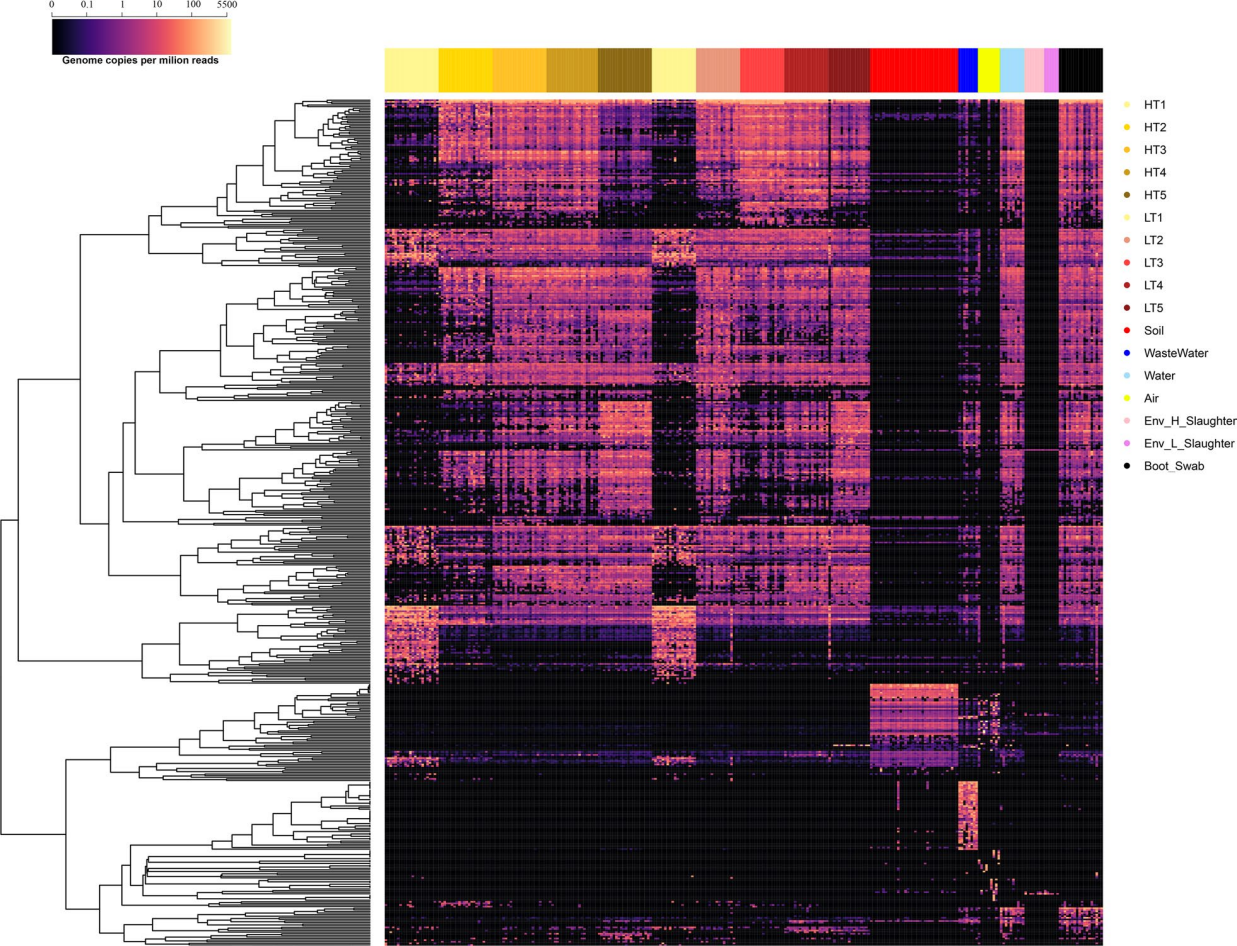
Distribution of all species-level genome bins across pig gut, farm environment, and slaughterhouse environment. Heatmap based on the abundance of species-level genome bins (SGBs) expressed as genome copies per million reads in each sample (grouped by color legend, top right). Samples include pig gut microbiome, soil surrounding the farm, air inside the farm, animal drinking water, wastewater, workers boot soles and slaughterhouse environment collected at different timepoints from the two farms (H & L). T1: farrowing unit, piglets from birth to 24 days; T2: start of the weaning phase; T3: end of the weaning phase; T4: start of the growing-finishing phase; T5: end of the growing-finishing phase

**Fig. 4:**
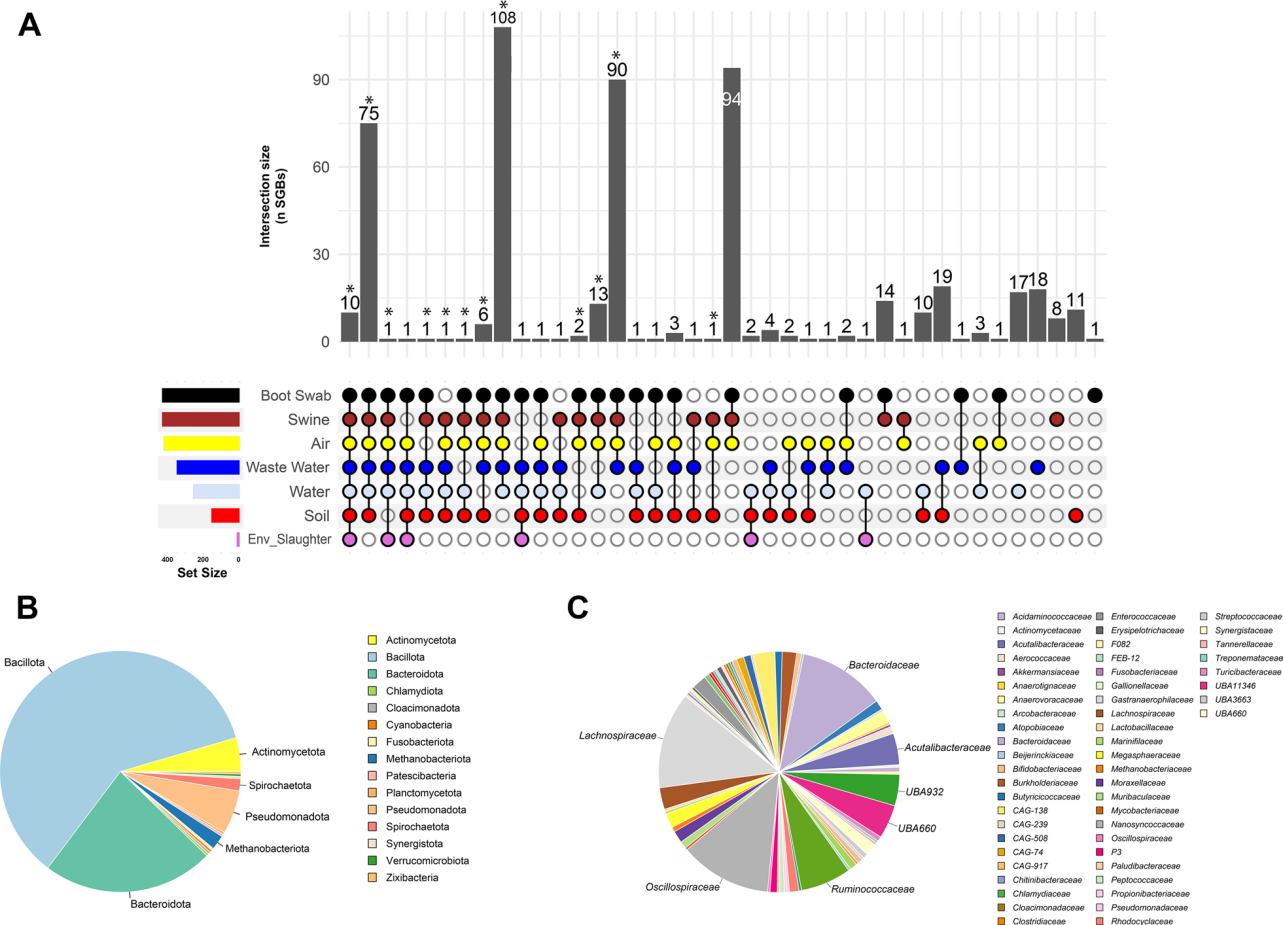
Sharing of species-level genome bins across pig gut and farm ecosystems with their taxonomic assignment. **A** UpSet plots showing the distribution of species-level genome bins (SGBs) across pig farm ecosystems (including pig gut, soil surrounding the farm, air inside the farm, animal drinking water, wastewater, workers boot soles and slaughterhouse environment). Taxonomic assignment of the 309 shared SGBs (highlighted in the UpSet plot by an asterisk) between pig gut, internal farm, and external farm ecosystems at the phylum (**B**) and family level (**C**)

We assessed the extent to which SGBs were dispersed across the pig fam system, including the animal gut and the internal/external farm environments and the slaughterhouse. To do this, we first mapped the metagenomic reads to our collection of 530 SGBs and then selected the shared species between the different farm ecosystems (Fig. 3). The SGB profile for each sample, expressed as genome copies per million reads, is shown in Additional file 4**: **Table S5. Three hundred nine out of the 428 SGBs being detected in the pig gut microbiome were widely distributed across the entire set of samples analyzed, including samples collected inside and outside the farm system (Fig. 4A). Most of these 309 SGBs (179, 60%) were assigned to the Bacillota phylum (Fig. 4B**)**. At the family level, 135 out of 309 SGBs (43%) were assigned to host-associated families, as *Lachnospiraceae* (40 SGBs), *Oscillospiraceae* (38 SGBs), *Bacteroidaceae* (36 SGBs) and *Ruminococcaceae* (21 SGBs) (Fig. 4C). Specifically, regarding the distribution of these pervasive 309 SGBs in the farm system, almost all of them have been detected in the internal farm environment, 308 and 307 SGBs being detected in farm air and boot soles samples as well, respectively. Interestingly, focusing on the external environments, we observed a differential distribution of the 309 pervasive SGBs, according to the different external ecosystems. In particular, 96 SGBs were detected in the external soil samples, 209 in watering places and 291 in the wastewaters. Finally, for each of the external ecosystem, we specifically accounted for the pervasive SGBs being assigned to well know host associated taxa, as taxa possibly deriving from the animal gut (Additional file 4**: **Table S6). Accordingly, in the external soil samples we have been able to detect 24 pervasive SGBs belonging to host associated taxa, 85 in the watering places and 132 in the waste waters, being the external farm ecosystem more contaminated by putative pig gut microbiome components. Finally, 11 core SGBs were detected as microbial species shared between almost all ecosystems, including the pig gut microbiome, the internal (boot soles and air) and external farm environment (soil, wastewater, and water from watering places) and including the slaughterhouse environment, highlighting the potential spread of these SGBs throughout the farm chain (only 1 was not shared with soil samples). These 11 SGBs were assigned to the genera *Propionicimonas*, *Syntrophosphaera, Methylocystis, Sideroxydans, Pseudomonas* (not identified at species level), and the species *Perlucidibaca aquatica, Bacteroides pyogenes, Escherichia coli, R.* SFHR01 sp004555545*, Novosphingobium* sp015657645 *and Acidovorax* sp001411535.

To increase the accuracy of the SGBs potential dispersion patterns across the farm system, we applied StrainPhlAn3 [54]. As StrainPhlAn3 works on single nucleotide polymorphisms, it was only possible to use the tool with the SGBs that were most represented in our samples. We therefore checked the strain sharing for those SGBs that had simultaneously at least 1 marker and verified that the same marker was present in at least 5 samples. According to our findings, a total of 281 unique SGBs were shared between pigs and the different environments considered in this study, some of which were shared between pigs and multiple environments (air, soil, wastewater). Specifically, 14 SGBs were recognized as the same strain shared between the pig gut microbiome and the internal or external farm environment (Additional file 1**: **Fig. S1). Of these 14 unique SGBs, 10 were found to be shared between pig and air microbiomes: *Clostridium* sp000435835*, Prevotella* sp002251435, *Mitsuokella jalaludinii*, *Bariatricus* sp004560705, *UBA2868* sp004552595 (family *Lachnospiraceae*), *Phil1* sp004558525 and *PeH17* sp004556165 (order Christensenellales)*, CAG-177* sp003514385 (family *Acutalibacteraceae*), *L. reuteri* and *UBA4334* sp900316975 (family *Bacteroidaceae*). Seven were shared between pig and wastewater microbiomes: *UBA1712* sp018056665 and *UBA2868* sp004552595 (family *Lachnospiraceae*), *C.* sp000435835, *Methanobrevibacter smithii*, *CAG-177* sp003514385 (family *Acutalibacteraceae*), *Turicibacter* sp001543345, *Sodaliphilus* sp004557565*.* Three were common to air, wastewater, and pig microbiomes: *C.* sp000435835*, CAG-177* sp003514385 (family *Acutalibacteraceae*) *and UBA2868* sp004552595 (family *Lachnospiraceae*). The normalized phylogenetic distance (nGD) values between the shared bacterial strains are reported in Additional file 4: Table S7 and S8.

### Dispersion of antimicrobial resistance determinants across the farm system

To assess the occurrence of antimicrobial resistance across the farm system, we first determined the presence of ARGs in the 309 previously detected pervasive SGBs, shared between the pig gut microbiome and the internal and external farm environments, 11 of which ben also deleted in slaughterhouse. For this purpose, we built a customized ARG catalog, based on the ORFs annotated as ARGs and retrieved from the assembled sequences using the PathoFact pipeline [9]. From a total of 5 million dereplicated ORFs (at 90% sequence similarity), 50,302 were assigned to ARGs and then refined to 682 ORFs, retaining only ORFs with a “strict” or “perfect” match (Additional file 3). Within the customized ARG catalog we identified 5% and 4% of the ORFs co-located respectively with phage and plasmid sequences, highlighting the potential of mobilityof these genes within our dataset.

**Fig. 5:**
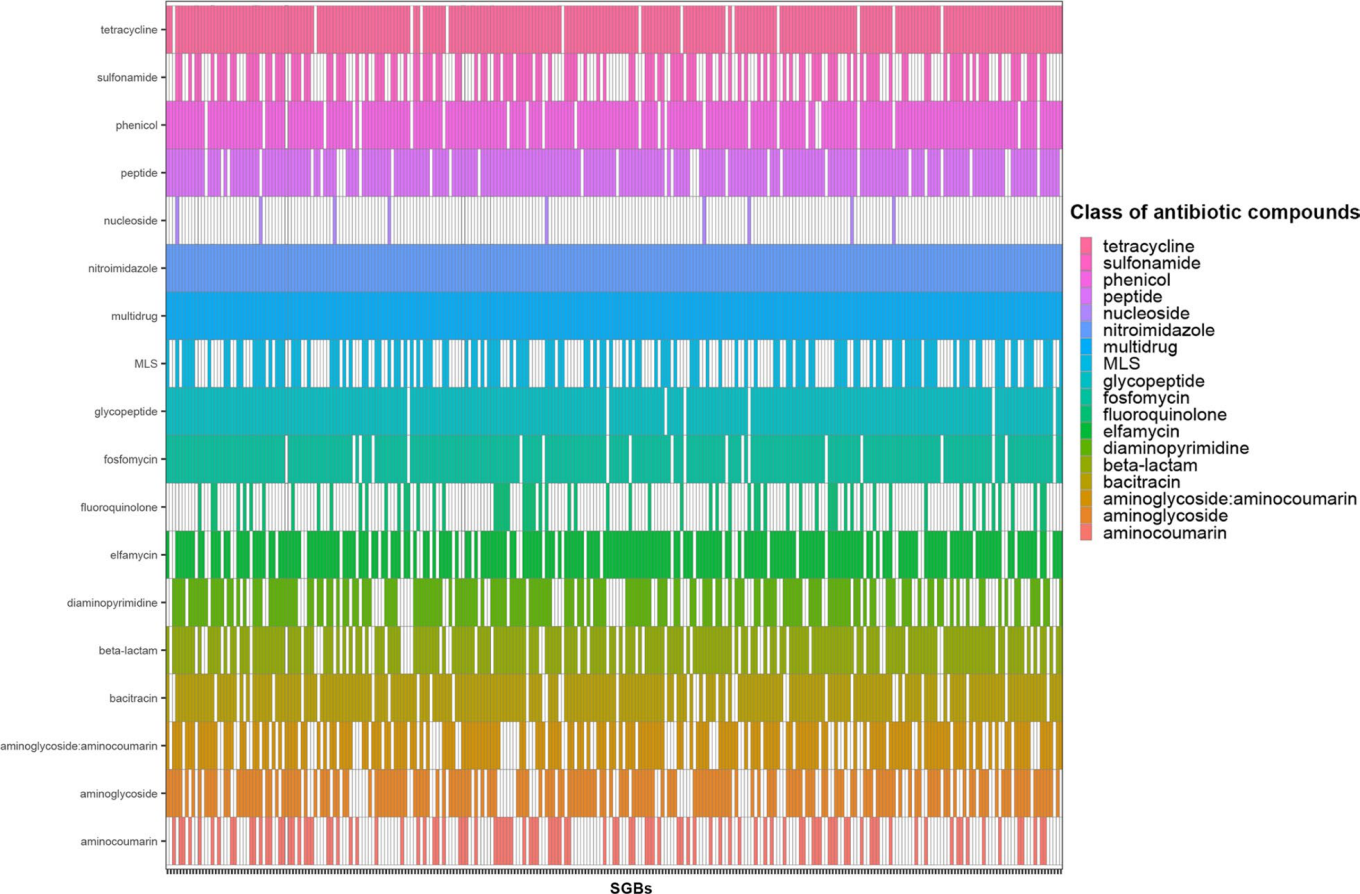
Occurrence of antibiotic resistance within the shared species-level genome bins. Presence/absence of antibiotic classes against which antibiotic resistance genes (ARGs) are active within the 309 species-level genome bins (SGBs) shared between pig gut, internal farm and external farm microbiomes. MLS: macrolide-lincosamide-streptogramin

**Fig. 6:**
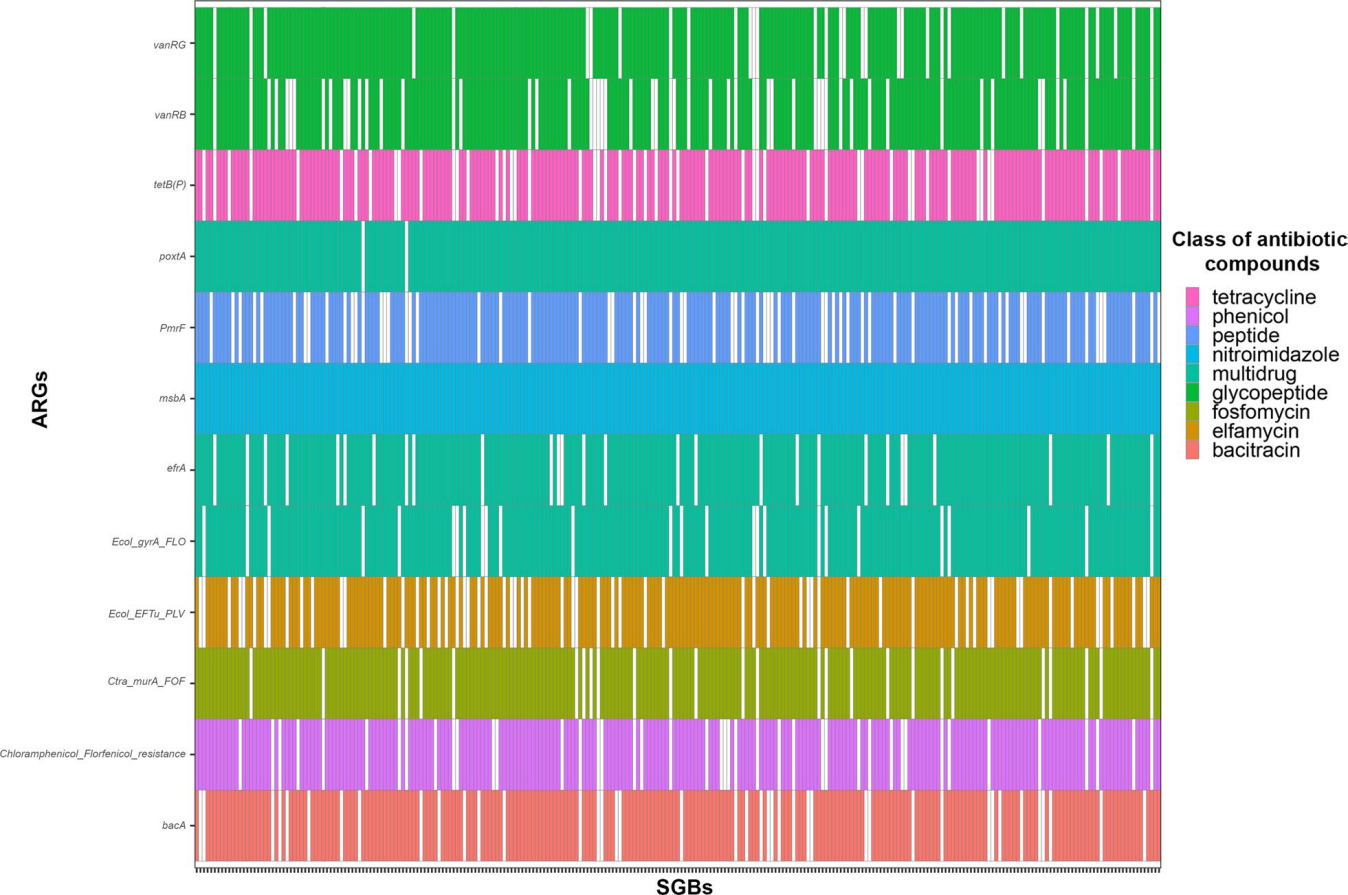
Occurrence of the core resistome within the 309 shared species-level genome bins. Presence/absence plot of antibiotic resistance genes (ARGs) showing a prevalence higher than 75% across the 309 species-level genome bins (SGBs) shared between pig gut, internal farm, and external farm ecosystems (classes of antibiotics against which antibiotic resistance genes are active are represented by color legend)

**Fig. 7:**
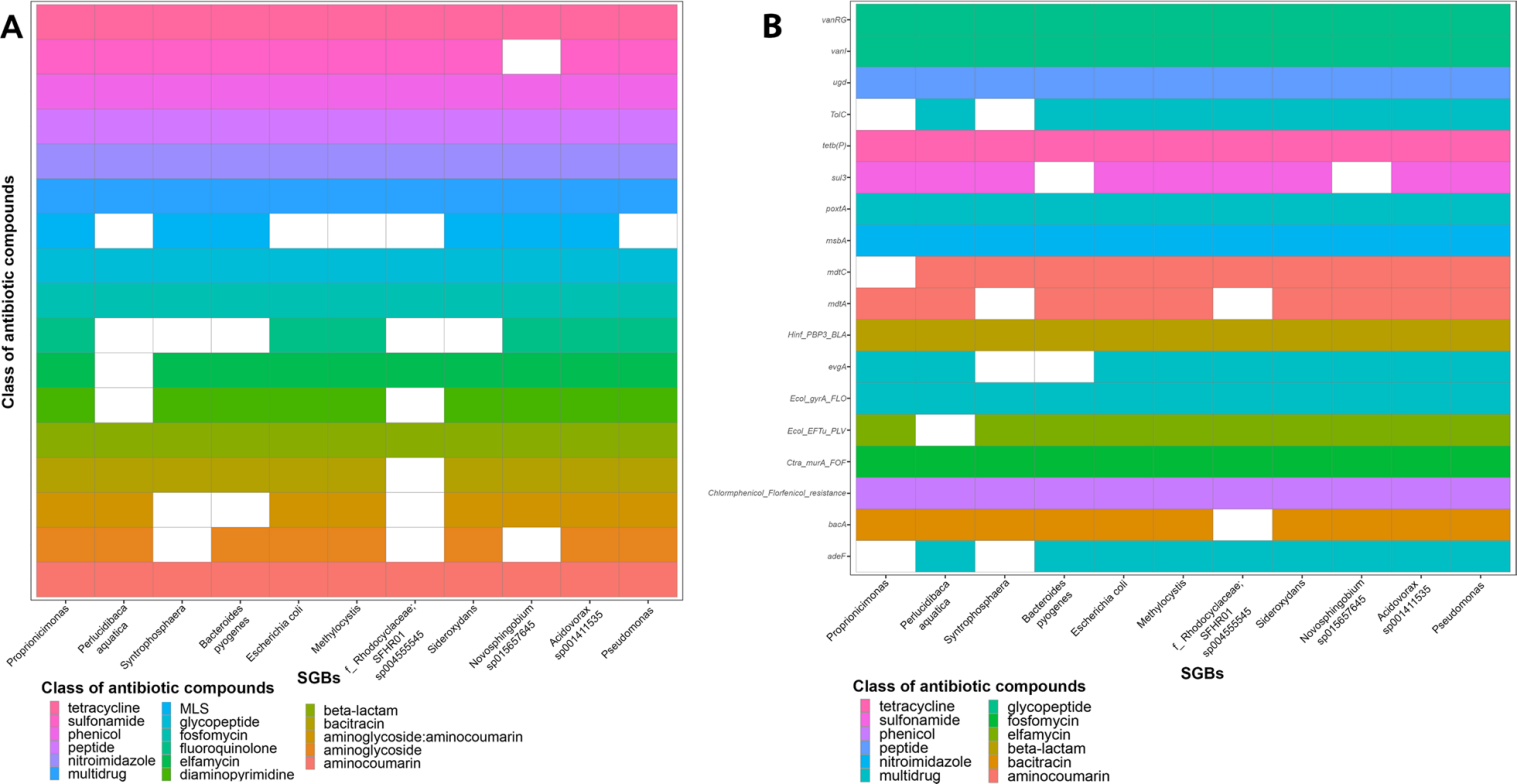
Occurrence of the core resistome within the 11 shared species-level genome bins. **A** Presence/absence plot of antibiotic classes against which antibiotic resistance genes (ARGs), within the 11 species-level genome bins (SGBs) shared between pig gut, internal farm, external farm, and slaughtering ecosystems, are active. **B** Presence/absence plot of ARGs showing a prevalence higher than 75% across the 11 SGBs, classes of antibiotics against which antibiotic resistance genes are effective are represented by color legend

We found that the 309 SGBs shared among the pig gut, internal farm (air or boot soles) and external farm (water, wastewater or soil) ecosystems contained 176 ARGs, which contributed to resistance against 18 different classes of antibiotic compounds (Fig. 5, Additional file 4: Table S9). In particular, resistance to nitroimidazole, multidrug (ARGs active against multiple antibiotic classes), glycopeptide antibiotics, tetracycline, phosphonic acid antibiotic (Fosfomycin), phenicol, antimicrobial peptide (general class and bacitracin), elfamycin, beta-lactam, amynoglicoside:aminocoumarin, diaminopyrimidine classes were the most represented (prevalence within the shared SGBs > 60%). On the other hand, resistance to other antibiotic compounds, such as macrolide-lincosamide-streptogramin (MLS), sulfonamide, aminocoumarin, fluoroquinolone, and nucleoside antibiotic classes, were the least common (prevalence within the shared SGBs < 50%; Fig. 5). In addition, 12 of the 176 ARGs were observed in at least 232 SGBs (75% of the shared SGBs), potentially representing the core resistome. This core was active against several antibiotic classes such as bacitracin (*bacA*), elfamycin (*Ecol_EFTu_PLV*), fosfomycin (*Ctra_murA_FOF*), glycopeptide antibiotics (*vanRG*, *vanRB*), multidrug (*efrA*, *Ecol_gyrA_FLO*), nitroimidazole (*msbA*), antimicrobial peptide (*PmrF*), phenicol (*Chloramphenicol_Florfenicol_resistance*), and tetracycline (*tetB(P)*) (Fig. 6). None of these core ARGs were predicted to be on plasmid or phage sequences. When focusing on the 11 SGBs also shared with the slaughterhouse environment, we found 107 ARGs conferring resistance to 17 different classes of antibiotic compounds (tetracycline, sulfonamide, phenicol, antimicrobial peptide, nitroimidazole, multidrug, MLS, glycopeptide, fosfomycin, fluoroquinolone, elfamycin, diaminopyrimidine, beta-lactam, bacitracin, aminoglycoside:aminocoumarin, aminoglycoside, and aminocoumarin). Interestingly, these antimicrobial resistances were highly represented in all 11 SGBs, with a prevalence > 50% (Fig. 7A), and 18 of the 107 ARGs were even more widely distributed (prevalence > 75%). These 18 genes were *vanRG*, *vanI* (conferring resistance to glycopeptides), *ugd* (resistance to antimicrobial peptides), *TolC*, *poxtA*, *evgA, Ecol_gyrA_FLO* and *adeF* (multidrug resistance), *tetB(P)* (resistance to tetracycline), *sul3* (resistance to sulfonamide), *msbA*, (resistance to nitroimidazole), *mdtC* and *mdtA* (resistance to aminocoumarin), *Hinf_PBP3_BLA* (resistance to beta-lactams), *Ecol_EFTu_PLV* (resistance to elfamycin), *Ctra_murA_FOF* (resistance to fosfomycin), *Chloramphenicol_Florfenicol_resistance* (resistance to phenicol) and *bacA* (resistance to bacitracin) (Fig. 7B). In particular, the vanRG ARG, a transcriptional activator of the OmpR-family, presented a variant annotated on a reference ORF co-located on a plasmid sequence. After the evaluation of the contigs carrying this ARG within the 11 SGBs, we highlighted that in 2 SGBs, the ARG was also co-located with a plasmid sequence, suggesting its possible horizontal mobility. Finally, when we verified the presence of these 18 ARGs in our samples, we found that they were present in 100% of the samples, proving their ubiquitous distribution, but they were generally present with a significantly lower abundance (in terms of RPKMs) in the soil and slaughterhouse environment compared to all other ecosystems (Wilcoxon rank-sum test, *p* < 0.05; Additional file 2**: **Fig. S2).

## Discussion

In this study, we longitudinally followed two pig production chains located in Italy from the suckling phase to the slaughterhouse to evaluate bacterial and ARG dispersion across the farm system, up to external environment. We found that several ARB (*i.e.*, SGBs) and ARGs circulation in the farm system, with a relevant fraction, possibly of animal origin, dispersing to the external environment.

Specifically, we generated 530 SGBs from 294 microbiome samples, spanning 7 different ecosystems (*i.e.*, pig gut microbiome, soil surrounding the farm, air within the farm, animal drinking water, wastewater, workers boot soles and slaughterhouse environment), with 367 SGBs assigned to previously characterized genomes and 163 (30%) representing new candidate species. When considering the SGBs dispersion pattern across the pig food system, 309 pervasive SGBs have been detected to be shared with both internal and external farm environment. Interestingly, these SGBs distributed differently across the external farm environments, with 24 SGBs being detected in the external soil samples, 85 in the watering places and 132 in the waste waters. Interestingly, among the 309 pervasive SGBs, 135 SGBs were belonging to host-associated taxa (e.g., *Oscillospiraceae*, *Lachnospiraceae*, *Bacteroidaceae* and *Ruminococcaceae*) [3], suggesting the possible animal origin. According to our finding, the great majority of these putative pervasive pig microbiome components were reaching waste waters (132 SGBs), while only 32 SGBs have been detected in the external soil. Taken together, these data suggest the contamination of waste waters as the possible main route of dispersion of host associated species from the farm system. Among the most prevalent pervasive SGBs of putative animal origin being detected in the wastewaters SGBs assigned to *Prevotella*, *Gemmiger* and *Blautia* genera have been detected. Finally, 11 pig gut components were defined as core SGBs, being detected in all ecosystems analyzed, including different surfaces of the slaughterhouse. Such SGBs included bacteria belonging to the genera *Propionicimonas*, *Syntrophosphaera, Methylocystis, Sideroxydans,* and *Pseudomonas* that were not identified at the species level, and the species *P. aquatica, B. pyogenes, E. coli, R. SFHR01 sp004555545, Novosphingobium sp015657645* and* A. sp001411535*. The genera *Syntrophosphaera* and *Pseudomonas* contain some bacterial species with anaerobic propensity, but in particular, one of the identified species, *B. pyogenes*, is a well-known anaerobic component of the pig gut microbiome.

Our data on the dispersion routes of SGBs across the pig farm system suggest a possible route of dispersion of pig gut components to surrounding environmental microbiomes, particularly trough the contamination of waste waters and raises important concerns about the spread of antimicrobial resistance, which can be rapidly transferred from pig gut components to the agricultural field, exposing farmers and rural residents to resistance determinants, with important implications for human health.

Indeed, according to our findings, the 309 SGBs shared between the pig gut microbiome and the external environment showed a diverse and complex resistome, with a structure well matched to the most commonly used antibiotics in the pig food chain in Europe (i.e. penicillins, third- and fourth generation cephalosporins, quinolones, aminoglycosides, polymyxins, and macrolides) [39, 41]. This confirms the relevant impact of antibiotic use in food-producing animal systems in shaping the gut resistome structure of farmed animals. Interestingly, among the resistance genes detected in these 309 SGBs, the vast majority conferred resistance to antimicrobial classes listed as “critically important or highly important” by the World Health Organization [47]. Specifically, 14 of the 18 ARGs belonging to the core resistome of the 309 SBGs and/or widely distributed (> 75%) in the 11 core SGBs, conferred resistance to antimicrobial classes defined as “critically important for human health”, such as *vanI, vanRG* and *vanRB* (conferring resistance to glycopeptide antibiotics), *PmrF* and *ugd* (resistance to antimicrobial peptides), *Ctra_murA_FOF* (resistance to fosfomycin compounds), *Hinf_PBP3_BLA* (resistance to beta-lactam compounds), and *TolC*, *msbA*, *poxtA*, *Ecol_gyrA_FLO*, *efrA*, *evgA* and *adeF* conferring resistance to multiple antibiotic classes. Indeed, resistance to these antibiotics has been found in pathogens of high clinical relevance, such as carbapenem-resistant *Acinetobacter*, *Enterobacteriaceae* and *Pseudomonas aeruginosa*, and vancomycin-resistant *Enterococcus faecium* [14, 15, 28]. In addition, one of these 14 ARGs was identified near to a plasmid sequence within two of the 11 widely occurrence SGBs. This ARG potentially mobile enclosed a transcriptional activator of the OmpR-family (*vanRG*), a response regulator that is part of a two-component regulatory system that determines vancomycin degradation [50, 64]. This suggests that this ARG can be mobilized between different bacteria by horizontal gene transfer, creating the conditions for the risk of transmission to clinically relevant bacteria in the worker's microbiome that are hazardous to human health. When we focused on the 11 core SGBs that can spread from the pig farm to the external environment and slaughterhouse, one of them was assigned to a multidrug-resistant *E. coli*, a pathogen that can colonize the pig and human gut [22] depending on the strain. This *E. coli*-assigned SGB was found to carry all ARGs present in the core resistome of the 11 shared SGBs, with some of these ARGs conferring resistance to antimicrobial classes defined as “critically important for human health”, such as glycopeptide antibiotics, antimicrobial peptides, fosfomycin compounds, and beta-lactam compounds. It is noteworthy that *E. coli* belongs to bacteria carrying ARGs defined by De Angelis et al. [8] with the acronym ESKAPEEc, which are responsible for a significant percentage of severe infections in hospitals (up to 75–80% of all bacterial isolates causing bloodstream infections) [11, 42]. Such ESKAPEEc list also includes *E. faecium*, *Staphylococcus aureus*, *Klebsiella pneumoniae*, *Acinetobacter baumannii*, *P. aeruginosa*, and *Enterobacter* [44]. Therefore, the presence of *E. coli* within the SGBs distributed in all ecosystems analyzed is a red flag for pig farm management and highlights the importance of monitoring the spread of resistance from the farm, especially that encoded by bacteria that are also pathogenic to humans.

## Conclusions

Overall, our study adds new details to the existing literature on the risk of ARGs and multidrug resistant bacteria dispersion in the farm system [16, 19, 40, 51]. The added value is the use of metagenomics and analysis of pig gut microbiome, together with the characterization of the microbiome from the soles of workers’ boots, air, wastewater, water and soil around farms. Monitoring the behavior of ARGs and multidrug resistant bacteria in the farm environments, including antibiotic resistant pathogens that pose a threat to human health, is even more important in pig farms, as pig manure and wastewater are commonly used as soil fertilizer, a procedure that could further increase the dispersion of these elements to the external environment. Subsequent research will prioritize the investigation of mobile elements to monitor the possible transfer of resistance determinants from commensal to pathogenic bacteria. Furthermore, an analysis of the worker's microbiome in relation to their farm exposure duration could be conducted in association with a longitudinal study could be designed to assess the impact of diverse production cycles over several years and to monitor the management practices concerning manure and discharges. These additional considerations could improve the assessment of the potential dispersion of bacterial taxa and their associated ARGs within the pig farm and beyond into the surrounding environment.

### Materials and methods

#### Animals, sample collection and processing

The trial was conducted in 1 commercial farm in the North Italy, with pigs raised in-door. Specifically, 96 piglets (17 days of age) from 24 litters were chosen from the same farrowing unit. At weaning (28 days of age), the piglets were equally allocated into two different weaning units (48 each) based on their body weight (BW) and litter of origin. These units were labeled as H and L. At the end of the weaning phase, piglets from weaning unit L were subsequently moved to the growing-finishing unit L, while piglets from weaning unit H were moved to the growing-finishing unit H.

Animal feces and surrounding environment were sampled during the rearing cycle, while at the slaughterhouse only the environment was sampled, for a total of 294 samples. Sampling was performed at five different timepoints between October 2019 and June 2020, as shown in Additional file 4: Table 1.

Specifically, a total of 199 feces were collected into two sterile 50-mL tubes by stimulating the rectum ampulla with sterile cotton swabs. The samples were then transported to the laboratory on dry ice. Soil was collected from 4 different areas surrounding the pig farm, at a depth of 10 cm using a 50-mL sterile tube [34], thus being able to collect the portion of soil where there was a higher probability of the presence of bacteria from the farm because the microbiome of soil surface it’s too much affected by atmospheric agents. Two liters of drinking water were collected in sterile bottles at the beginning of the water pipe at each timepoint. The water was transported to the laboratory and filtered onto cellulose mixed ester 0.22 µm pore-size filters (MF-Millipore, Darmstadt, Germany) through a vacuum filtration system. Bioaerosol was collected by sampling the air within the farm using a pump connected to a filter, set at a flow rate of 2–8 L/min over approximately 6 h. The filter was then removed and placed in a sterile tube. From the manure lagoon, 4 samples of wastewater were collected using a sterile 50-mL tube. Overshoe samples were collected using a sterile swab rubbed onto the operators’ boots after walking inside the pig farm. Finally, 40 samples from slaughterhouse environment were collected through sterilize swabs. In particular, 20 samples for each pig production chain were sampled from conveyor belt surfaces, floor and slaughterhouse walls. All samples were immediately transported to the laboratory and stored at -80 °C if not processed immediately. All sampled categories with the corresponding quantity are reported in Additional file 4: Table S2.

#### DNA extraction and shotgun metagenomic sequencing

DNA was extracted using different commercial kits depending on the matrix source, namely the DNeasy PowerWater kit (Qiagen, Hilden, Germany) for water, the FastDNA SPIN Kit for Soil (MP Biomedicals, Santa Ana, Ca, USA) following the manufacturer’s instruction for feces, and the DNeasy PowerSoil kit (Qiagen) for all other samples.

A total of 294 samples (199 pig fecal samples, 36 soil samples, 10 air samples, 9 water samples, 8 wastewater samples, 18 boot swab samples, 14 slaughterhouse environmental samples resulting from the pooling of the 40 collected swabs based on the environmental origin) were collected and processed for shotgun metagenomic sequencing. DNA was quantified using the QUBIT fluorimeter (Invitrogen, Waltham, MA, USA) and DNA libraries were prepared using the QIAseq FX DNA library kit (Qiagen). Briefly, DNA samples were fragmented to a 450/500-bp size, end-repaired, and A-tailed using the FX enzyme mix and the thermal cycle instructions provided by the manufacturer. DNA samples were then incubated with DNA ligase and Illumina adapter barcodes for 15 min at 20 °C to perform adapter ligation. A purification step using Agencourt AMPure XP magnetic beads (Beckman Coulter, Brea, California, USA) was followed by a 10-cycle PCR for samples containing less than 100 ng of DNA. DNA libraries were additionally purified and then pooled at an equimolar concentration of 4 nM. Final libraries were sequenced on an in-house Illumina NextSeq platform, located at the University of Bologna sequencing facility, using a 2 × 150 bp paired-end protocol to obtain > 3 Gb per sample.

#### Species-level genome bins (SGBs) definition and ARG identification

Reads were filtered following the standard operative procedures of the Human Microbiome Project [53], adapting the procedures to pig samples when necessary.

As a first operation step, host DNA was removed from the raw reads of the pig samples using the bmtagger software, with the *Sus scrofa* genome as a reference (RefSeq assembly accession: GCF_000003025.6). Reads were then quality trimmed, ensuring a minimum quality score of 20, and length truncated using trimBWAstyle [52]. Duplicates were identified and eliminated using the Picard tool EstimatedLibraryComplexity (v. 1.71). Next, high-quality reads were assembled using megahit (v. 1.2.9) with default settings. The resulting assembly files from the pig samples were utilized to create a curated gene catalog using the PathoFact pipeline (v 1.0) [9], the first version of the catalog was additionally revised using the RGI tool (v. 6.0.2) [1] to retrieve only open reading frames (ORFs) with “perfect” and “strict” matches, based on the CARD’s curated bit-score cut-offs automatically computed by the tool. Additionally, the metawrap binning module (metawrap v. 1.3.2) was employed to construct MAGs from each sample. Only MAGs with completeness over 50% and contamination lower than 5% were retained, assessed through the checkm lineage_wf workflow [37]. Further refinement was carried out by dereplicating the high-quality MAGs into species-level genome bins (SGBs) using the dRep dereplicate command (v. 3.2.2) with the following parameter “–ignoreGenomeQuality -pa 0.9 -sa 0.95 -nc 0.30 -cm larger -centW 0”. Taxonomic classification of SGBs was performed using the gtdbtk classify_wf workflow with default parameters (Chaumeil et al., 2020). The abundance of SGBs in each sample was quantified using the metawrap quant_bins module (metawrap v. 1.3.2) and to annotate the genomes, prokka (v. 1.14.6) was employed using the previously curated ARG catalog described above, as the priority database for annotation. For unannotated ORFs, prokka (v. 1.14.6) uses additional databases such as UniProtKB and hidden Markov model profiles, including combined_Toxin, dbCAN-fam-HMMs, HAMAP, and Virulence_factor. Finally, all GFF files generated by prokka were concatenated and the presence or absence of annotated ORFs in each SGB was determined using Roary with the parameters -i 90 -cd 25 -e -g 1,000,000. The PathoFact annotation of each gene cluster calculated by Roary was extracted from the concatenated GFF file using the locus_tag reference in the presence/absence table. This presence/absence table of the ARGs within the SGBs was also used to assess the abundance of ARGs within the samples, by multiplying it with the matrix of the SGBs abundance across samples. Also, the co-localization of *vanRG* gene with plasmid sequences was assessed using PlasFlow tool (v. 1.1) [24] with default parameters, retrieving the full contigs sequences of the 11 core SGBs where the ARG was found.

Additionally, the SGBs obtained were compared, using MinHash sketches implemented in the mash tool (v. 2.3), with 4930 SGBs from > 150,000 human gut microbiome MAGs, including different individuals, spannning age, geography, and lifestyle [38] and a total of 7489 genomes from two of the most recent studies based on MAGs regarding the pig gut microbiome [6, 20, 23], to understand if they were already known identified genomes.

#### Detection of strain-sharing events

To gain a deeper understanding of the potential sharing of microbiome components between pig and environmental metagenomes, we performed a strain-level population structure analysis using StrainPhlAn3 [2] as previously described in the work by Valles-Colomer et al. [56]. Specifically, we performed the analysis on the most abundant SGBs, i.e. those that are represented by at least 5 MAGs and whose abundance was > 5 GCMs in at least one pig individual. Then, we constructed a custom SGB marker database for each species under investigation. This was done by selecting core genes specific to each SGB from the output of the Roary tool. Core genes were defined as genes present exclusively in the examined SGB and absent in the rest of the dataset. Then MAGs within each SGB were fragmented into 150-nucleotide fragments and aligned against their corresponding core genes using bowtie2 (v. 2.3.4.3) with the "–sensitive" option. A core gene was considered a valuable marker gene for an SGB if it was mapped by at least 90% of the MAGs, covering more than 50% of the gene’s length.

To investigate strain sharing, strain-level phylogenies were reconstructed using bowtie2 (–sensitive option) and StrainPhlAn3, with the parameters "–marker_in_n_samples 1 –phylophlan_mode accurate". Additionally, a parameter called "–sample_with_n_markers" was set to retain only samples with at least 10 marker genes.

To identify instances of strain sharing, we first set SGB-specific normalized phylogenetic distance (nGD) thresholds. These thresholds effectively separated the distributions of nGD values for strains retained within the same pig group (indicating the same strain) from those of different pig group (indicating different strains) in our pig samples. The nGD values were calculated as leaf-to-leaf branch lengths normalized by the total tree branch length in the phylogenetic trees generated by StrainPhlAn3, which were constructed based on marker gene alignments. The nGD thresholds were defined by maximizing Youden's index and limiting at 5% the fraction of unrelated individuals to share the same strain as a bound on a false discovery rate.

#### Biostatistics

All statistical analyses were performed using the R software (v. 4.2.0, www.r-project.org) with the packages *vegan* (v. 2.6-2) [36], RcppAlgo (v. 2.6.0) [63], xlsx (v. 0.6.5) [13], ggVennDiagram (v. 1.2.2) [18], ggplot2 (v. 3.4.0) [61], ComplexUpset (v. 1.3.3) [31], RColorBrewer (v. 1.1-3) [35], gplots (v. 3.1.3) [58], viridis (v. 0.6.2) [48], reshape2 (v. 1.4.4) [60], tidyverse (v. 1.3.2) [62], and hrbrthemes (v. 0.80) [43]. The vegdist function (method = "bray”) was used to calculate beta diversity while the diversity function was used to calculate alpha diversity. Both functions are included in the vegan package. Separation of data in principal coordinate analysis (PCoA) was assessed using a permutation test with pseudo-F ratios (adonis function in the vegan package). The Wilcoxon rank-sum and Kruskal–Wallis tests were used to assess differences in alpha diversity and ARGs abundance (in terms of RPKMs) distributions between groups. *P*-values were adjusted using p.adjust by method = "fdr" of the function in R. Adjusted *p*-values ≤ 0.05 were considered statistically significant.

### Supplementary Information

**Additional file 1: Fig. S1**. Schematically representation of bacterial strains cooccurrence across pig feces and other environments. Colored dots represent SGBs identified as the same strain between samples. As highlighted, three strains are in common with more than one environment and pig feces. The others are exclusively shared between pig gut microbiome and only one environmental sample.

**Additional file 2: Fig. S2**. Abundance of the 18 shared ARGs within all ecosystems. Description of data: Boxplots based on the abundance of the 18 shared ARGS, expressed as genome copies million reads, all ARGs showed a significant variation in terms of abundance among all microbial ecosystems (Kruskal-Wallis test, p-value < 0.001). Sample groups marked with different letters are statistically significant different (Wilcoxon rank-sum test, p < 0.05).

**Additional file 3**. Antibiotic resistance genes catalog. Antibiotic resistance genes open reading frames within our metagenome samples.

**Additional file 4**. Supplementary Tables S1–S9.

## Data Availability

High-quality reads from all samples are deposited in the European Nucleotide Archive under the project accession number PRJEB68161. SGBs are available here: https://site.unibo.it/microbiome-science-biotechnology-unit/en/microbiome-materials-and-databases.
