# Supplementary figures and images for: Dispersion of antimicrobial resistant bacteria in pig farms and in the surrounding environment
Source: Anim Microbiome. 2024 Mar 30;6:17. doi: 10.1186/s42523-024-00305-8 (PMC10981832; doi:10.1186/s42523-024-00305-8)

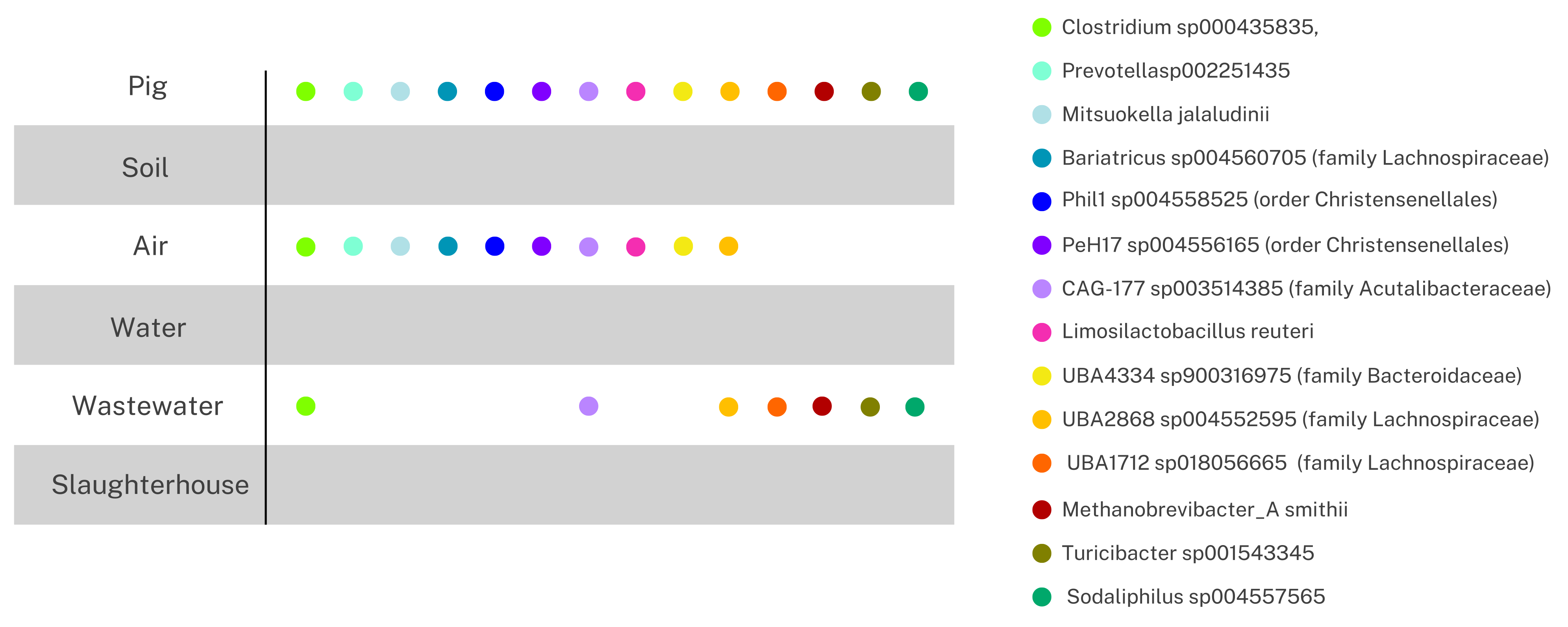

Supplement: Supplementary file 1 — Additional file 1: Fig. S1. Schematically representation of bacterial strains cooccurrence across pig feces and other environments. Colored dots represent SGBs identified as the same strain between samples. As highlighted, three strains are in common with more than one environment and pig feces. The others are exclusively shared between pig gut microbiome and only one environmental sample. [file 42523_2024_305_MOESM1_ESM.png]

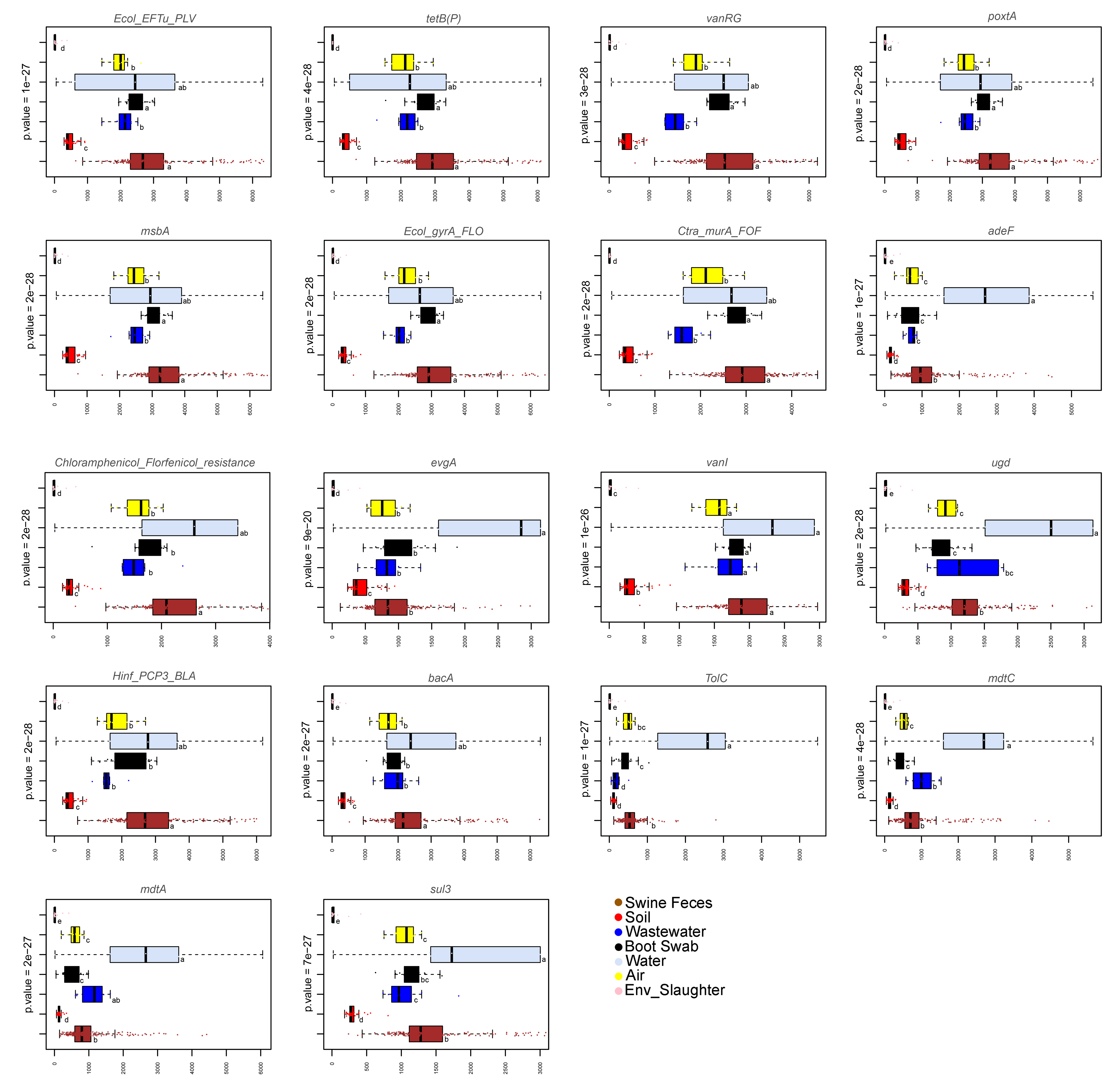

Supplement: Supplementary file 2 — Additional file 2: Fig. S2. Abundance of the 18 shared ARGs within all ecosystems. Description of data: Boxplots based on the abundance of the 18 shared ARGS, expressed as genome copies million reads, all ARGs showed a significant variation in terms of abundance among all microbial ecosystems (Kruskal-Wallis test, p-value < 0.001). Sample groups marked with different letters are statistically significant different (Wilcoxon rank-sum test, p < 0.05). [file 42523_2024_305_MOESM2_ESM.png]
